# Supplementary material for: Experiences of family caregivers in caring for patients with heart failure admitted at Jakaya Kikwete Cardiac Institute, Dar es Salaam, Tanzania: A qualitative study
Source: PLoS One. 2023 Sep 20;18(9):e0280698. doi: 10.1371/journal.pone.0280698 (PMC10511101; doi:10.1371/journal.pone.0280698)
Supplement: S1 File — (DOCX) [file pone.0280698.s001.docx]

## INTERVIEW GUIDE ENGLISH FORM.

TITLE OF THE STUDY: EXPERIENCE OF FAMILY CAREGIVER ON CARING FOR A PATIENT WITH HEART FAILURE AT JAKAYA KIKWETE CARDIAC INSTITUTE.

Interview site……………………….. Interviewee No …………………………

Date………………………………… Start time………………………………

End time……………………………..

PART A: INTERVIEWEE DEMOGRAPHICS

Age: ………………………………………. Sex: …………………………………………

Marital status ……………………………… Education ……………………………………

Occupation ………………………………………………….

Relation to the patient ………………………………………

PART B: Needs of caregivers

1. What are the needs of people who take care of patient with HF? Which skills are you supposed to develop in caring for patient with heart failure? In which ways do you build your skills?
2. How do you communicate with healthcare professionals especially nurses regarding caring for your patient with HF? How does this help you in caring for your patient with HF?
3. How does caring of your patient with HF affect your sleeping pattern?
4. How does caring for patient with HF affect your own health care needs?

PART C: Challenges faced by caregivers in caring for patients with heart failure

1. What challenges do you face in about caring for patient with heart failure?
2. What activities do you do, apart from caring for your patient with heart failure? How do they affect your participation in caring for your patient?
3. How do you divide your time for social interaction and for attending your patient with heart failure?
4. What support do you receive in caring for your patient with HF? Who provide you with support? Is the support sufficient?

PART D: Support from nurses

1. What support do you get from nurses when it comes to caring for your patient with heart failure? How does the support received help you emotionally, financially or socially?
2. What instructions from nurses do you receive on handling your patient with HF? How does this help you in caring for HF patient? How is it beneficial to you and your patient with heart failure?
